# TSHR in thyroid cancer: bridging biological insights to targeted strategies

**DOI:** 10.1530/ETJ-24-0369

**Published:** 2025-07-03

**Authors:** Shaojie Xu, Youyun Peng, Xingyin Li, Hanning Li, Ting Liu, Xingrui Li, Yaying Du

**Affiliations:** ^1^ Department of Thyroid and Breast Surgery, Tongji Hospital, Tongji Medical College, Huazhong University of Science and Technology, Wuhan, Hubei, People’s Republic of China; ^2^ Union Hospital, Tongji Medical College, Huazhong University of Science and Technology, Wuhan, Hubei, People’s Republic of China; ^3^ Department of Anesthesiology, Tongji Hospital, Tongji Medical College, Huazhong University of Science and Technology, Wuhan, Hubei, People’s Republic of China

**Keywords:** TSHR, differentiated thyroid cancer, radioiodine-refractory differentiated thyroid cancer, targeted strategies

## Abstract

Traditionally, thyroid-stimulating hormone receptor (TSHR) has been utilized primarily to increase the efficacy of radioactive iodine therapy by promoting iodine uptake. However, the rise of personalized medicine has prompted reassessment of the potential of TSHR as a therapeutic target. Recent studies have indicated that TSHR plays a critical role in the progression of thyroid cancer and may serve as a key target for the treatment of residual or metastatic thyroid cancers, particularly radioiodine-refractory differentiated thyroid cancer (RAIR-DTC). This review focuses on the biological characteristics of TSHR and its potential as a therapeutic target, emphasizing that optimizing TSHR-targeted drugs and integrating them with existing treatment strategies could offer new therapeutic avenues for patients with RAIR-DTC.

## Introduction

Over the past decade, the incidence of thyroid cancer has significantly increased (1, 2). Differentiated thyroid cancer (DTC), which accounts for 96% of cases, is typically treated with surgery, followed by thyroid-stimulating hormone (TSH) suppression therapy and radioactive iodine therapy (3, 4). Most patients with DTC have a favorable prognosis after standard therapeutic approaches. However, the risk of local recurrence and distant metastasis may reach as high as 20 and 10%, respectively, posing significant challenges for diagnosis and treatment (5). Among these patients, two-thirds may progress to radioiodine-refractory differentiated thyroid cancer (RAIR-DTC), which has a poor prognosis, with a 10-year survival rate of only approximately 15% (5). RAIR-DTC is generally considered resistant to chemotherapy. Although current targeted therapies can prolong progression-free survival, they are significantly limited by high rates of drug resistance, notable adverse effects, and a fundamental inability to fully eradicate tumors (6). Current strategies for eradicating RAIR-DTC have inherent limitations, highlighting the need for new technologies to improve treatment outcomes.

Thyroid-stimulating hormone receptor (TSHR) is a large transmembrane glycoprotein consisting of 764 amino acid residues and belongs to the G protein-coupled receptor superfamily regulated by guanine nucleotides (7). Under normal physiological conditions, TSH binds to TSHR to maintain the normal growth and proliferation of thyroid cells, regulate iodine metabolism, and mediate the synthesis and secretion of thyroid hormones. Through the adenylyl cyclase signaling pathway, TSHR participates in critical processes such as energy metabolism and growth (8, 9).

The unique role of TSHR in DTC and its potential as a therapeutic target make it a subject of intense research. Exploring the biological functions of TSHR and its mechanisms in DTC is crucial for developing novel therapeutic strategies. This review systematically summarizes the biological characteristics of TSHR in DTC, related signaling pathways, and the potential for targeted therapies, aiming to provide new insights and directions for future research and clinical applications.

## Expression and clinical significance of TSHR

TSHR is widely expressed in extrathyroidal tissues such as adipose tissue, extraocular muscles, kidneys, liver, vascular smooth muscle, thymus, adrenal glands, red blood cells, and immune cells (10, 11, 12, 13, 14, 15, 16, 17). The role of TSHR expression outside the thyroid is not completely understood. In contrast to the situation in thyroid cancer, high expression of TSHR in other tumor types is closely associated with malignant progression and poor prognosis, likely due to distinct activation states and regulatory pathways in these tumors. TSHR is expressed in both normal ovarian tissue and human epithelial ovarian cancer cells and functions through the TSH-TSHR pathway (18). Overactivation of TSHR can promote the proliferation of ovarian cancer cells by modulating G protein-coupled signaling pathways and indirectly activating epithelial growth factor receptors (19). TSHR is expressed in almost all melanocytic lesions, with higher expression observed in malignant and precancerous lesions (20). In addition, TSHR has been confirmed to participate in liver metabolism regulation (21, 22). Its overexpression is prevalent in the majority of hepatocellular carcinoma tissues and is linked to poor prognosis (23). Similarly, TSHR is significantly overexpressed in breast cancer and thymic malignancies (24, 25). The role of TSHR in extrathyroidal tissues is worthy of investigation.

The expression level of TSHR in thyroid tissue is several hundred times greater than that in other tissues (26). Immunohistochemistry (IHC) typically reveals strong positivity for TSHR in DTC (26, 27). However, early studies suggested that the actual amount of TSHR on the cell surface was very low, with approximately 10^3^–10^4^ receptors expressed per thyroid follicular cell (28). In some cases of DTC, TSHR mRNA levels are low despite elevated protein expression, suggesting that the discrepancy may result from posttranscriptional regulation, altered protein stability, or differential translation efficiency (29). A comprehensive assessment of the absolute expression level of TSHR on the cell surface of patient-derived thyroid cancer cells is essential for understanding its potential role in tumor progression and therapeutic targeting.

TSHR expression varies significantly across different tumors (30), which suggests heterogeneity across different cancer cell populations. Approximately 85% of RAIR-DTC tumors and 75% of metastatic lymph nodes exhibit TSHR expression (26), with heterogeneous expression levels across different metastatic lymph nodes (31). In addition, residual or recurrent DTC has been shown to respond to exogenous TSH stimulation (32), indicating that TSHR remains present in most clinically significant DTC metastases, even after radioiodine treatment. A decreasing trend in TSHR expression has been observed in advanced and poorly differentiated DTC (30). TSHR suppresses thyroid cancer cell invasion and metastasis by inhibiting epithelial–mesenchymal transition (EMT) in thyroid cancer cells (33). Studies assessing the differences in TSHR levels on KAT-5 cells revealed that downregulation of TSHR expression weakly inhibited thyroid cancer cell proliferation but significantly increased metastasis (34).

Dedifferentiation is a common feature of thyroid cancer and is often accompanied by a decrease in TSHR expression, which is typically associated with increased tumor invasiveness and poor prognosis. TSHR, as part of the thyroid differentiation score, is used to quantify the differentiation status of thyroid cells (35, 36). TSHR can promote iodine uptake in thyroid cells under physiological conditions and can also upregulate sodium/iodide symporter (NIS) mRNA expression and regulate its targeted transport to the cell membrane (37). TSHR gene transfection contributes to the redifferentiation of dedifferentiated thyroid follicular carcinoma cells (38, 39). This redifferentiation, along with the subsequent increase in radioactive iodine uptake, may be associated with activation of the TSHR/cAMP signaling pathway (40).

## Structure of the TSHR protein

The TSHR protein comprises three domains: the extracellular, transmembrane, and intracellular domains (41). The extracellular domain contains 418 hydrophilic amino acids, including a leucine-rich repeat (LRR) domain and a hinge region (42). These regions are closely involved in the binding of TSHR with TSH secreted by the pituitary gland; in particular, the α helix formed by the LRR, the horseshoe structure formed by β chains, and the sulfated tyrosine 385 residue at the C-terminus of the hinge region directly affect TSH binding (43, 44). In addition, the TSHR extracellular domain includes six highly conserved glycosylation sites, which contribute approximately 30–40% of the receptor’s molecular weight through N-glycosylation (45). This glycosylation is crucial for maintaining receptor stability and function, facilitating TSHR localization to the cell membrane, and enhancing TSH binding by prolonging the receptor residence time. TSH binding induces an upright active conformation in the extracellular domain of TSHR, which then triggers conformational changes in the transmembrane domain, ultimately activating the intracellular G protein signaling pathway (46, 47). The hinge region of TSHR not only connects the LRR and transmembrane domains but also directly regulates ligand binding and signal transduction. Mutations or deletions in the 371–384 residue region increase constitutive activity (48).

The transmembrane domain, which consists of seven helical segments, constitutes the principal structural element responsible for mediating signal transduction in TSHR (49). TSHR shares high sequence homology in the transmembrane domain with other pituitary glycoprotein hormone receptors (such as the luteinizing hormone receptor and the follicle-stimulating hormone receptor), but the homology in the extracellular domain is lower (50). More than 15 cholesterol molecules surround TSHR, assisting its localization to lipid rafts and thereby increasing signal transduction efficiency (51, 52). However, mutations at positions 58 and 209 of the TSHR protein, such as lysine substitutions, can significantly reduce TSH binding affinity, resulting in a more than tenfold decrease in signal transmission efficiency (47).

## Signal transduction of TSHR

**Figure 1:**
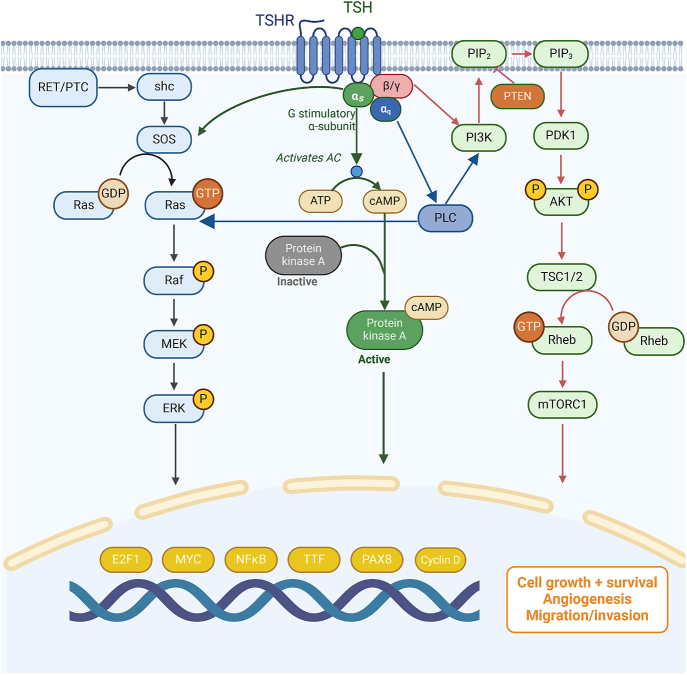
Signaling pathways mediated by TSHR.

TSHR couples with G proteins through its intracellular domain and primarily initiates signal transduction via the Gs protein. Activation of the Gs pathway increases the abundance of cyclic adenosine monophosphate (cAMP), which activates protein kinase A (PKA). PKA phosphorylates downstream molecules, regulating enzymes and genes such as thyroid peroxidase (TPO) and the sodium/iodide symporter (NIS), thereby promoting thyroid hormone synthesis and secretion (53). In addition to the Gs pathway, TSHR also signals through the Gq protein, which activates phospholipase C (PLC) to hydrolyze PIP2 into inositol trisphosphate (IP3) and diacylglycerol (DAG) (53). DAG and PKA can both stimulate protein kinase C (PKC), which, in turn, activates the AKT pathway, influencing cell survival, proliferation, growth and glycogen metabolism (54). TSHR signaling is also functionally linked to the MAPK/ERK cascade. cAMP elevation can activate ERK1/2 through both PKA-dependent and alternative intermediates such as Rap1, while PKC – activated via the Gq pathway – can also facilitate ERK phosphorylation through the Ras-Raf axis (53). These converging signals reflect the complex crosstalk among the Gs, Gq, and MAPK pathways downstream of TSHR (Fig. 1). Moreover, β-arrestins bind to activated TSHR, preventing further G protein coupling and terminating the classical signaling cascade (55). They also promote TSHR internalization and may activate noncanonical pathways, such as MAPK/ERK signaling (56).

There are also some new perspectives on the signal transduction of TSHR; aberrant TSHR signaling pathways can promote angiogenesis and the malignant proliferation of cells by stimulating the secretion of vascular endothelial growth factor and C-X-C motif chemokine ligand 8 (57). Activation of the TSH-TSHR signaling pathway has also been shown to regulate thyroid cancer cell migration and differentiation via the PI3K/AKT/mTOR signaling pathway (58). In thyroid cancer and gliomas, the TSH-TSHR regulatory axis, particularly high TSHR expression, has been found to facilitate immune evasion by tumor cells (59). TSHR inhibitors have been shown to suppress tumor immune evasion by inhibiting programmed death-ligand 1 (PD-L1) expression and activating T effector cells (59).

## Detection of TSHR in thyroid cancer

The use of clinical biomarkers for thyroid cancer evaluation has certain limitations. For example, serum Tg, an effective marker for the postoperative surveillance of thyroid cancer patients, has reduced sensitivity during TSH suppression therapy and experiences interference from Tg autoantibodies (60). TSHR expression is significantly positively correlated with the degree of differentiation in thyroid carcinoma (61). Accurate detection of TSHR expression enhances staging and follow-up assessments.

TR1401 is a synthetic TSH analog designed with four lysines added to its structure; this modification increases its affinity and signal transduction activity with specific negatively charged residues in the hinge region of TSHR, making TR1401 a ‘superagonist’ (62). Upon labeling with the radionuclide 99mTc, TR1401 has strong imaging capabilities in TSHR-positive cell lines, TSHR-positive thyroid tumor models, and poorly differentiated DTC models (63). Lysine residues in TR1401 are replaced with arginine residues to create TR1402 for a further increase in affinity for TSHR, particularly in thyroid cancer cell lines (64). Conjugation of TR1402 with deferoxamine and zirconium-89 results in improved tumor uptake, accelerated blood clearance, and significantly reduced off-target accumulation, making TR1402 a promising noninvasive biomarker for DTC (65, 66). In contrast to agonists such as TR1401 and TR1402, which preferentially bind the high-affinity state of TSHR, TSHR antagonists can bind to the receptor in its low-affinity state. Exploiting this feature, the TSHR antagonist K1-70 monoclonal antibody has been radiolabeled with Zr-89, creating a specific TSHR imaging agent (67). This radiotracer is particularly useful in detecting TSHR with low expression or in inactive states, suggesting novel diagnostic potential for RAIR-DTC. While not yet in clinical use, this application expands the utility of PET imaging in thyroid cancer diagnosis. In summary, TSHR-targeted imaging and therapeutic agents demonstrate high specificity and sensitivity for thyroid cancer diagnosis. However, challenges remain, including cost, radiation exposure, and potential immune responses. Ongoing optimization and preclinical trials are crucial to advancing these methods toward broad clinical application.

mRNA expression detection provides direct quantification of TSHR levels but requires high sample quality and RNA stability to ensure accuracy (68, 69). Liquid biopsy, which measures circulating TSHR mRNA in blood, enables noninvasive and real-time monitoring and is particularly useful when tissue samples are inaccessible (70). Digital PCR excels in detecting low-abundance mRNAs, significantly reducing false positives and negatives, and is suitable for the precise quantification of TSHR levels (71). IHC offers a visual representation of the TSHR distribution within tissue and is cost-effective, but it is limited to fixed tissue samples and provides lower quantitative accuracy (72).

## TSHR research models

Studies investigating the expression levels of TSHR in thyroid cancer cell lines have revealed that many of these cell lines no longer express TSHR but instead resemble undifferentiated thyroid carcinoma (73, 74, 75). This property may be due to the early dedifferentiation of cells during monolayer culture, which results in the downregulation of TSHR expression (76). An inconsistency in TSHR expression data for FTC-133 cell lines derived from lymph node metastases of differentiated follicular thyroid carcinoma across different laboratories has been noted, which may be attributable to genetic drift or epigenetic changes occurring during cell line passaging (77, 78). Similarly, conflicting reports on TSHR expression in BCPAP cell lines have been published (78, 79). Upon adapting the culture conditions to better mimic the human physiological environment, the follicular thyroid carcinoma cell line FTC-238, derived from a lung metastasis, exhibited a marked upregulation of TSHR expression, whereas no such change was observed in 8305C cells (80). The XTC-UC1 cell line derived from metastatic Hǔrthle cell carcinoma retains the expression of TSHR and other differentiation markers, but it is less widely used because of its genetic instability and metabolic characteristics, which may not accurately represent broader thyroid cancers (75, 81).

*In vitro* TSHR-targeted studies commonly use stable TSHR-transfected cell lines. However, the expression levels of TSHR in these cell lines may not accurately reflect TSHR expression in patients in the real world, and TSHR expression itself shows heterogeneity across populations. Therefore, to enhance the clinical translatability of this research, it is necessary to either perform absolute or relative quantification of TSHR expression or to employ primary cell or organoid models. Patient-derived thyroid cancer organoids have potential as personalized preclinical models that faithfully mimic parental tumor biology *in vitro* and are useful for studying clinical responses to anticancer drugs (82). Evaluating TSHR expression in thyroid cancer organoids holds promise for addressing the issue of TSHR loss in cell lines. Sondorp *et al.* developed a patient-derived thyroid cancer organoid model, and although similarities in TSHR expression were observed between papillary thyroid cancer (PTC) tissue and organoids, the expression levels of PAX8, TG, c-MET, and TSHR decreased in thyroid cancer organoids during passaging, suggesting the dedifferentiation of these organoids during propagation (83). Recent studies have developed a PTC organoid model derived from mouse embryonic stem cells (mESCs) by introducing the BrafV637E mutation (84). However, the activation of BrafV637E leads to a time-dependent downregulation of TSHR mRNA expression. Notably, the combined inhibition of the MEK and PI3K pathways not only restores TSHR expression levels but also facilitates the re-expression of thyroid functional markers and the structural recovery of the organoids. The combination of TSHR-targeted therapy with MAPK and PI3K pathway inhibitors may offer a novel approach for the treatment of thyroid cancer patients with low TSHR expression.

## Targeted therapy based on TSHR

**Figure 2:**
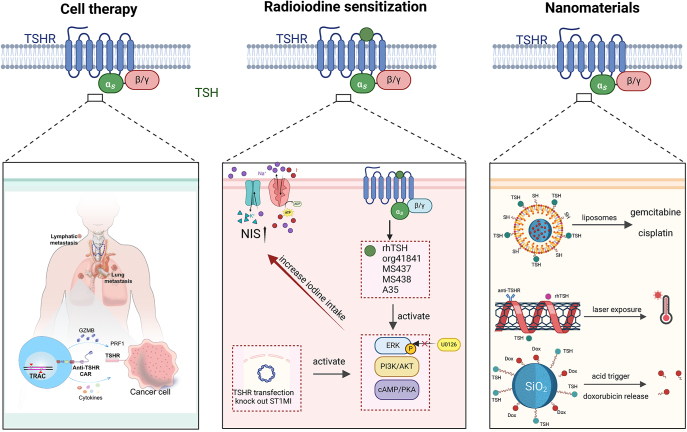
Overview of TSHR-targeted therapeutic strategies, including cell therapy, radioiodine sensitization, and nanomaterials.

Targeted therapy has led to revolutionary changes in cancer treatment (85). The precise delivery of drugs to specific tissues effectively reduces side effects, expands the therapeutic window, and increases drug efficacy (86). Patients with recurrent or metastatic thyroid cancer often require aggressive treatments that are associated with numerous adverse effects (85). TSHR, a key regulator of thyroid function, has now become a critical target in the treatment of DTC. TSHR-based therapeutic approaches, including small-molecule drugs, nanomaterials, and advanced immunotherapies, represent new avenues for treating thyroid cancer, particularly in patients who have developed resistance to traditional treatments, such as radioiodine therapy (Fig. 2).

### Small molecule drugs

Compared with peptide and protein drugs, chemically synthesized small molecules offer advantages such as rapid membrane permeability, oral bioavailability, low-cost synthesis, and ease of large-scale production, making them more suitable for drug development (87). Significant efforts have been made to identify novel small molecules that target TSHR for potential application in the treatment of DTC.

Radioiodine remains the most effective treatment for thyroid cancer after surgery, with reduced expression of NIS being the primary reason for resistance to radioactive iodine therapy (88). Approximately 10–15% of DTCs lose NIS expression and take up iodine but retain TSHR expression (89). Currently, before radioiodine therapy, recombinant human thyroid-stimulating hormone (rhTSH) is administered or L-T4 is discontinued to increase TSH levels, thereby increasing the uptake of radioactive iodine by thyroid cancer cells (90). However, discontinuing L-T4 may lead to hypothyroidism, and rhTSH was therefore developed to provide TSH stimulation without withdrawing thyroid hormones and has been approved as an adjunct in diagnostic procedures for DTC patients. Unfortunately, rhTSH is costly, with challenges in maintaining stable supplies of high-quality materials, which has prompted the academic community to strive to identify cheaper and more reliable TSHR agonists. The thiophenyl compound org41841 has been identified as a partial agonist of TSHR that acts through binding to the transmembrane domain of the receptor (91). However, its ability to activate TSHR is too low for clinical application. Building on the structural features of org41841, researchers developed the first high-affinity, selective TSHR agonist, NCG00161870, which effectively activates TSHR and stimulates thyroid function in human thyrocytes and in mouse models (92). It increases the mRNA expression of NIS, thereby increasing radioactive iodine uptake in the thyroid. Latif and colleagues utilized a luciferase-cAMP high-throughput screening system to screen 50,000 compounds and identified two novel TSHR agonists, MS437 and MS438 (93). These lead compounds exhibit favorable pharmacokinetic profiles and are capable of increasing both NIS and TSHR gene expression.

TSHR agonists have the dual effects of increasing the efficacy of radioactive iodine therapy and potentially accelerating tumor progression; moreover, even after TSH stimulation, 10–20% of DTC fails to concentrate sufficient radioactive iodine for effective treatment (29). Therefore, the use of TSHR agonists should be closely monitored and typically combined with radioactive iodine treatment. In contrast, the recently reported novel orally active TSHR-targeting ligand A35 effectively inhibits human thyroid cancer proliferation and metastasis both *in vitro* and *in vivo* as a standalone treatment, showing minimal toxicity and strong potential as a lead compound for DTC therapy (94).

### Cell therapy

Chimeric antigen receptor T (CAR-T) cell therapy, a revolutionary type of cancer immunotherapy, has shown significant efficacy in the treatment of hematologic malignancies (95). In recent years, researchers have gradually explored its potential application in solid tumors (96). CAR-T-cell therapy for advanced thyroid cancer has focused primarily on ICAM-1 as a target (97, 98). However, owing to the expression of ICAM-1 on immune and endothelial cells, targeting ICAM-1 carries the potential risk of off-target toxicity and associated side effects (99).

TSHR-targeted CAR-T-cell therapy was first reported in 2020 at the ASCO Annual Meeting and demonstrated significant efficacy in two RAIR-DTC patients, with one achieving complete remission and the other achieving near complete remission (100). Sanders and Evans *et al.* reported that three human-derived monoclonal antibodies targeting TSHR, K1–18, and M22, exhibit thyroid-stimulating activity, whereas K1–70 has blocking activity (101, 102). On the basis of these three antibodies, Li *et al.* developed three third-generation CAR-T cells. Owing to the superior performance of K1-70 CAR-T cells in terms of expansion capacity, survival rate, and phenotypic stability, they conducted a comprehensive evaluation of their efficacy and safety in the treatment of DTC (26). Previous studies have shown that 4-1BB domains are linked to reduced toxicity, enhanced T-cell persistence, and a more controlled peak of T-cell expansion, whereas CD28 costimulatory domains are associated with faster onset of CAR-T-cell activity but with faster subsequent exhaustion (103). Similar conclusions have been drawn in studies targeting TSHR. Compared with CAR-T cells with 4-1BB domains, CAR-T cells with CD28 domains may exhibit a greater response rate and cytotoxicity upon antigen recognition, potentially because of ERK activation (104). Ding *et al.* demonstrated that TSHR-targeted CAR-T cells effectively recognized and killed TSHR-expressing thyroid cancer cells, releasing IL-2, IFN-γ, TNF-α, and Granzyme-B (105). On the basis of these findings, a patient with recurrent, refractory thyroid cancer was recruited and received TSHR + CD19 CAR-T-cell therapy; this patient achieved partial remission within 3 months and tolerated the treatment well.

Compared with CAR-T-cell therapy, natural killer (NK) cell therapy offers unique advantages, including a lower incidence of cytokine release syndrome and graft-versus-host disease, as well as shorter production times and reduced costs (106). Recently, Zhou’s team validated the safety and efficacy of anti-TSHR CAR-engineered NK cell therapy for DTC in both cell and animal models (107). These engineered NK-92 cells demonstrated significantly enhanced cytotoxicity against TSHR-positive DTC cell lines, along with increased degranulation and cytokine release, offering promise as a new option for advancing immunotherapy for DTC. The overview of cell therapy research is provided in Table 1.

**Table 1:** Overview of cell therapy research.

Time	Research category	Cell therapy types	Costimulatory molecule	Source of ScFv	Reference
2020	Clinical study	CAR-T	-	-	(100)
2022	Preclinical study	3GEN CAR-T	4-1BB and CD28	K1-70; M22; K1-18	(26)
2022	Preclinical study	2GEN CAR-T	4-1BB vs CD28	K1-70; KSAb	(104)
2022	Clinical and preclinical study	2GEN CAR-T	4-1BB	-	(105)
2024	Preclinical study	CAR-NK	4-1BB	M22	(107)

3Gen, third generation; 2GEN, second generation.

### Nanomaterials

Nanoparticle-based cancer therapy is intended to circumvent the pharmacokinetic limitations and drug resistance of traditional treatments while minimizing damage to systemic and adjacent normal tissues (108). With sole reliance on the enhanced permeability and retention (EPR) effect, accumulation in tissues occurs nonspecifically because of the lack of precision in selectively targeting the desired regions (109). To overcome these limitations, engineered nanomaterials targeting tumor-specific antigens have emerged as a potentially powerful approach in cancer treatment (110). By conjugating nanomaterials with targeting ligands, active targeting to specific cells is achieved, thereby reducing toxicity to nontarget cells (111). Building on this research paradigm, researchers have begun to explore the application of novel nanomaterials that target TSHR in DTC.

Liposomes, a mature nanodelivery strategy, were initially assessed as carriers for chemotherapy drugs targeting TSHR. Encapsulating chemotherapeutic drugs such as gemcitabine or cisplatin in TSH-linked liposomes can significantly increase their intracellular uptake efficiency in TSHR-expressing cells (77, 112). Following intravenous injection in mice, targeted nanoliposomes accumulate in the thyroid at 3.5–4 times higher levels than nontargeted nanoliposomes do. A plausible explanation is that binding to TSHR to form cross-linked clusters decreases the Gibbs free energy at the contact site, inducing the internalization of liposomes and thereby enhancing the ability of liposomes to target and penetrate DTC cells (113). Photothermal therapy is suitable for superficial tumors such as thyroid cancer and can effectively overcome the resistance of thyroid cancer to traditional chemotherapy drugs (114). Multiwalled carbon nanotubes with photothermal conversion effects can be surface functionalized through covalent or noncovalent interactions with various chemical groups, proteins, and small molecules, thereby increasing their targeting ability in biological systems (79). pH-sensitive release technology leverages the acidic tumor microenvironment to release drugs precisely at the tumor site, thereby increasing therapeutic indices and reducing systemic toxicity (115). Li *et al.* developed silica TSH-SiO_2_/Dox nanoparticles that trigger the release of doxorubicin in acidic tumor microenvironments. These nanoparticles can target thyroid cancer cells expressing TSHR through interaction with TSH, increasing treatment specificity and reducing the cardiotoxicity of doxorubicin (116).

In addition to TSH, commercial TSHR antibodies and rhTSH can also be used as targeting molecules in engineered nanomaterials (79). However, the high cost and limited editability of antibodies restrict their widespread application in the development of nanomaterials (117). Therefore, identifying optimized alternatives is crucial. Aptamers, synthetic nucleic acids composed of single-stranded DNA or RNA, offer advantages such as high affinity, low cost, and excellent editability (118). Compared with antibodies, aptamers are easier to synthesize and exhibit significantly reduced batch-to-batch variability. Cao *et al.* developed a TSHR-targeting aptamer, TSHR-21-42, which effectively competes with antibodies for binding sites on TSHR and facilitates cellular uptake following binding (119). Although TSHR-21-42 was initially designed for thyroid-associated ophthalmopathy, it holds great potential as a tool for TSHR-targeting nanomaterials. The overview of TSHR-targeted nanomaterials is presented in Table 2.

**Table 2:** Overview of targeted drug delivery systems.

Time	Targeting molecule	Loaded drug	Nanomaterials	Reference
2014	TSH	Gemcitabine	Liposomes	(78)
2015	TSH	Cisplatin	Liposomes	(112)
2016	TSHR Ab/rhTSH/TSH	Photothermal therapy	Multiwalled carbon nanotubes	(80)
2017	TSH	Dox	SiO2	(116)

Ab, antibodies.

Some *in vitro* studies have shown that rhTSH does not significantly induce proliferation or migration in papillary thyroid carcinoma cell lines (120). There are nevertheless concerns that *in vivo*, nanomaterials containing TSH, the natural agonist of TSHR, may promote the proliferation and dissemination of thyroid cancer cells through mechanisms such as activation of the cAMP pathway. In the treatment of DTC, the use of TSHR inhibitory antibodies or other inhibitors, such as TSHR inverse agonists, as ‘navigators’ for nanomaterials has significant therapeutic potential. These ‘navigators’ not only target DTC cells for efficient drug delivery but also may block cancer cell proliferation by inhibiting the TSHR signaling pathway while maintaining high binding affinity (94).

To date, the inclusion and exclusion criteria for TSHR-targeted therapies have not been clearly defined. However, on the basis of existing treatment strategies and the current understanding of TSHR, it can be inferred that TSHR-targeted therapy may be applicable to the following patients: i) patients with recurrent, refractory thyroid cancer who have undergone complete resection of thyroid tissue; ii) patients in whom TSHR is expressed positively only in thyroid cancer or its metastatic lesions and is not expressed in other major organs; and iii) patients who did not respond to I^131^ or sorafenib. The specific indications and contraindications for different treatment approaches still need to be further clarified through clinical trials and research. With further research advances, TSHR-targeted therapies are expected to complement radioiodine therapy, offering novel strategies for systemic treatment in the postoperative setting and improving the long-term prognosis of DTC patients.

## Challenges and future prospects

Although TSHR is critical for maintaining thyroid function and physiological activities, abnormally expressed or mutated TSHR can also activate multiple signaling pathways, which leads to the abnormal proliferation and differentiation of thyroid cells and tumorigenesis. The persistent expression of TSHR in DTC makes it an ideal candidate for targeted thyroid cancer therapy, with particular potential in the treatment of RAIR-DTC. However, the activation status and regulatory pathways of TSHR may vary among different patients (121, 122). Therefore, the biological functions of TSHR in tumorigenesis and development must be clarified to develop a TSHR-targeted thyroid cancer therapeutic strategy. Conducting multiomics studies and multidisciplinary collaborations may accelerate the development of TSHR-targeted therapy. Moreover, the application of targeted therapeutic drugs necessitates increased attention to individual characteristics. Thus, detecting individualized levels of TSHR and their changes in thyroid tumors is crucial for developing therapies and determining patient prognosis. The development of precise and sensitive methods, such as oligonucleotide probes or microarrays, to assess TSHR levels for the design of reasonable and personalized treatment plans has emerged as crucial in the treatment of thyroid-related diseases, particularly in cancer therapy.

CAR-T cells targeting TSHR effectively kill TSHR-positive tumor cells and inhibit tumor metastasis (26, 100). However, on the basis of the limited clinical trials and data available, systematic assessments of the efficacy and safety of TSHR-targeted cellular therapy are currently difficult. Moreover, preventing antigen escape by tumor cells and addressing issues such as cytokine release syndrome and neurotoxicity in CAR-T-cell therapy remain critical challenges. To improve outcomes, combining CAR-T-cell therapy with immune checkpoint blockade or having CAR-T cells express certain enzymes that degrade the tumor stroma may enable CAR-T cells to traffic to and infiltrate solid tumors in the immunosuppressive tumor microenvironment by crossing physical tumor barriers (123). Furthermore, the production processes of CAR-T cells and methods used to produce off-the-shelf and universal CAR-T cells that target TSHR also appear to be important factors influencing the development of TSHR-targeted CAR-T-cell therapy. *In vivo* gene editing via CAR-T-cell technology may reduce the production risks and time costs associated with TSHR-targeted CAR-T cells. Other emerging cell therapies have also shown unique advantages. For example, CAR-NK cell therapy has a lower risk of immune rejection and strong innate killing ability (107). Chimeric antigen receptor macrophage (CAR-M) therapy not only directly targets and kills tumor cells but also reshapes the tumor microenvironment, thereby increasing the durability of the immune response (124). The development of TSHR-targeted CAR-M-cell therapy provides a powerful complement to TSHR-directed cell therapies.

Although many nanomaterials have achieved good tumor delivery efficiency in animal experiments, most of them have failed to achieve the desired effect in clinical trials. This difference could be because the absorption, biodistribution, metabolism, and excretion properties of the nanomaterials may differ between animals and humans. Thus, models that can imitate the complex conditions within human bodies must be established to reflect the properties of nanomaterials. After nanomaterials enter the body, their interactions with biological molecules, cells, and tissues determine their fate, such as whether they can accumulate in target areas or trigger systemic toxicity. On the basis of this consideration, machine learning techniques can be employed to optimize the design of nanomaterials, such as by assisting with surface modification and functionalization and predicting their behavior in biological systems (125). Furthermore, correcting abnormal gene expression in tumor cells is a trend in targeted therapy for tumors, and the successful application of liposomes for mRNA and siRNA delivery has made nanomaterial-based gene editing for regulating TSHR possible in thyroid cancer therapy (126). Thus, strengthening interdisciplinary cooperation, such as combining biology, medicine, and materials science, is crucial for accelerating the application of nanomaterials in TSHR-targeted thyroid tumor therapy.

The concept of using TSHR-targeted antibody–drug conjugates and radioimmunotherapy for the treatment of refractory DTC has long been proposed but has yet to achieve practical progress. The primary reason for the difficulty is the conflict between the complexity and high cost of development and the uncertainty of efficacy (44). Current TSHR-targeted therapy has shown potential in treating refractory DTC, but in resource-limited areas, its high cost, complex production process, and reliance on specialized medical resources limit its economic feasibility and accessibility. Therefore, cost reduction and efficiency improvement are key factors to consider during the development process. Despite challenges and a lengthy industrialization process, ongoing research and technological development will ultimately make this a reality.

## Declaration of interest

The authors declare that there is no conflict of interest that could be perceived as prejudicing the impartiality of the work reported.

## Funding

This work was supported by the Key Research and Development Program of Hubei Province (Grant No. 2022BCA007), the National Natural Science Foundation of Chinahttps://doi.org/10.13039/501100001809 (No. 82203392), the Knowledge Innovation Program of Wuhan-Shuguang Project (Grant No. 2023020201020490), and the Bethune Charitable Foundationhttps://doi.org/10.13039/100016966 (No. 2117).

## Author contribution statement

SX and YP contributed to the design and literature review of the manuscript. XL reviewed the manuscript content. HL participated in data validation and interpretation. TL assisted with the literature screening and provided technical support. XL and YD provided project supervision and manuscript revisions. All the authors read and approved the final manuscript.

## Data availability

All of the material is owned by the authors, and is available upon reasonable request to the corresponding author.
